# Management of angle closure glaucoma

**DOI:** 10.4103/0301-4738.73690

**Published:** 2011-01

**Authors:** Jovina L S See, Maria Cecilia D Aquino, Joel Aduan, Paul T K Chew

**Affiliations:** 1 Department of Ophthalmology, National University Health System, National University Hospital, Singapore; 2 Department of Ophthalmology, National University Health System, National University of Singapore, Singapore

**Keywords:** Acute attack glaucoma, laser iridoplasty, laser iridotomy, primary angle closure, primary angle closure suspect, primary angle closure glaucoma

## Abstract

Primary angle closure glaucoma (PACG) is equally prevalent in Indian in Asian population as the primary open angle glaucoma. Eighty-six percent of people with PACG are in Asia, with approximately 48.0% in China, 23.9% in India and 14.1% in southeast Asia. To understand PACG, it is mandatory to understand its classification and type of presentation with the underlying pathophysiology. The treatment options are medical, laser and/or surgical. The present article provides an overview of PACG.

Glaucoma is ranked as the leading cause of irreversible blindness worldwide by the World Health Organization.[1] It has been estimated that 3.9 million people with glaucoma will be blind due to primary angle closure glaucoma (PACG) by 2010. By 2020, this number is projected to increase to 5.3 million.[2] Eighty-six percent of people with PACG are in Asia, with approximately 48.0% in China, 23.9% in India and 14.1% in southeast Asia.[2] These numbers highlight the importance of understanding the disease, its natural history, and its underlying pathophysiology, so that we may try to establish effective methods of treatment and preventative measures to delay, or even arrest, disease progression, thereby reducing visual morbidity.

Patients with primary narrow angle may be classified as a primary angle closure suspect (PACS), or as having primary angle closure (PAC) or primary angle closure glaucoma (PACG) [Box 1].[3]

**Box 1:** Classification Based on Natural History

**Primary Angle Closure Suspect:** An eye in which appositional contact between the peripheral iris and posterior trabecular meshwork is present or considered possible, in the absence of elevated intraocular pressure (IOP), peripheral anterior synechiae (PAS), disc or Visual Field (VF) changes. Epidemiologically, this has been defined as an angle in which 180–270º of the posterior trabecular meshwork cannot be seen gonioscopically.
**Primary Angle Closure:** PACS with statistically raised IOP and/ or primary PAS, without disc or VF changes.
**Primary Angle Closure Glaucoma:** PAC with glaucomatous optic neuropathy and corresponding VF loss.
Based on objective findings, this classification is widely used in the classification of subjects in research and has been adopted in the Asia Pacific Glaucoma Guidelines. However, patients within each group can present differently and warrant different management. The traditional classification of PACG based on clinical presentation [Box 2] is more helpful to the clinician in allowing easy recognition of the type of management necessary.

Management of patients with PAC depends on the type of clinical presentation, making the diagnosis of PACS, PAC or PACG, as well as correctly identifying the underlying pathophysiology. Treatment options may be medical, laser and/or surgical.

## Medical Management

**PACS:** Patients assessed to be at risk of angle closure (AC) warrant prophylactic laser peripheral iridotomy. Prior to laser therapy, a parasympathomimetic like pilocarpine is helpful to induce pupil miosis and iris stromal thinning so that laser may be more easily performed. The α_2_-agonists like brimonidine work quickly to lower IOP and may be used prior to and/or after laser peripheral iridotomy to prevent an IOP spike. Topical steroids instilled four times daily for a week after laser are beneficial in reducing post-laser intraocular inflammation.

**Acute AC:** Immediate medical therapy in acute AC consists of commencing IOP-lowering eye medications such as topical β-blocker, α_2_-agonist and even prostaglandin analogues as soon as possible. Once the IOP is sufficiently reduced to allow iris reperfusion, pilocarpine is instilled to induce miosis in an attempt to widen the anterior chamber angles and reestablish aqueous outflow. (Pilocarpine should, however, be avoided in cases where it may exacerbate pupil block, such as in pseudoexfoliation, phacomorphic glaucoma and aqueous misdirection, i.e., where AC is secondary to lens-induced or retro-lenticular mechanisms.) In addition, intravenous or oral acetazolamide 5–10 mg/kg [alternatives: hyperosmotic agents, e.g., intravenous 20% mannitol 1–2 g/kg, oral 50% glycerol 1–1.5 g/kg (contraindicated in diabetics), oral isosorbide 1.5–2.0 g/kg] is often useful in helping to lower the IOP and hastening resolution of corneal edema so that a laser peripheral iridotomy can be definitively done. Topical steroids help to reduce intraocular inflammation, while analgesics and anti-emetics help make the patient more comfortable, until laser peripheral iridotomy is done (see section on Lasers).

**Chronic PAC/PACG:** Once the patient has been treated with laser peripheral iridotomy (and laser iridoplasty where indicated), long-term medical treatment including topical β-blockers, α_2_-agonists and carbonic anhydrase inhibitors can be used if IOP control remains suboptimal. Recent studies have demonstrated that prostaglandin analogues such as latanoprost, bimatoprost and travoprost are also effective in lowering IOP in chronic PACG, even in the presence of 360° of PAS.[5–10]

## Laser Management

### Laser peripheral iridotomy

Laser peripheral iridotomy [Fig. 4] is the current standard approach to initial treatment of AC. It alleviates pupillary block, which is a common underlying mechanism of AC [Box 3]. Evidence indicates that laser peripheral iridotomy is probably useful in the early stages of PACG, but once extensive synechial angle closure and glaucomatous optic neuropathy have developed it is less likely to be effective in lowering IOP.[11] Argon and neodymium (Nd):yttrium-aluminum-garnet (YAG) lasers are widely used when performing laser peripheral iridotomies. A suitable combination of power and time of exposure appropriate to the iris thickness and degree of iris pigmentation is critical to produce the desired effect.[12] Nd:YAG laser is useful in helping to further enlarge the opening in the iris.[13] This method of sequential argon–Nd:YAG laser peripheral iridotomy is very effective, especially in thick, dark irides that are otherwise difficult to perforate with either one of the lasers alone. With this sequential technique, the total mean energy used has been reported to be as little as a third of the energy used in pure argon and Nd:YAG iridotomies.[14] The sequential technique also minimizes the incidence of iris bleeding.

**Box 2:** Classification Based on Clinical Presentation

**Acute:** Sudden onset of IOP elevation resulting from total angle closure, accompanied by symptoms of severe, usually unilateral, ocular pain, red eye, blurred vision, haloes, headache (ipsilateral frontal), nausea and vomiting.
**Subacute/Intermittent/Creeping:** An episode of sudden IOP elevation that is spontaneously aborted, so that symptoms are mild or even absent. Such subacute IOP elevations may be recurrent and therefore termed “intermittent angle closure”. Intermittent episodes can result in progressive PAS formation, termed “creeping angle closure”.
**Chronic:** Chronic IOP elevation due to the presence of PAS that permanently close the anterior chamber angle. Symptoms are usually absent.
**Latent:** Evidence that an open but narrow angle can and does close under certain circumstances. Asymptomatic, but PAS is often found on gonioscopy.
Although helpful, both forms of classifications above do not identify the pathophysiological mechanism underlying the angle closure. A classification devised by Ritch and colleagues[4] is useful for this purpose and should be used in parallel in order to further facilitate the clinician in choosing an appropriate treatment [Box 3].

**Table:** 

**Indications:[15]**	PACG	
	PAC	
	PACS, especially if:	Presence of PAC in fellow eye
		Family history of ACG
		Need for repeated dilated examinations
		Poor access to regular ophthalmic care
**Procedure:[15]**	Topical pilocarpine to miose pupil and stretch (thin) iris.	
	Abraham/Wise iridotomy lens with coupling fluid	
	Choose iris crypt or an area of thin iris. Avoid level of tear meniscus formed by lid and globe. Aim at peripheral iris, avoiding any areas of corneal arcus senilis.	
	Nd:YAG 2–5 mJ, 1–3 pulses/burst	
	Argon laser 700–1100 mW, 50 µm spot size, 100 ms, 10–20 burns can be used prior to Nd:YAG in a thick iris to photocoagulate and thin the iris stroma, thereby also reducing the risk of iris bleeding.	
	*Endpoint:* Iris pigment plume, lens visible through iridotomy, laser iridotomy (LI) size of about 150–200 µm. Brown Asian irides are thicker than blue ones and may require a larger iridotomy where there is intraocular inflammation.	
**Complications:[15]**	Corneal endothelial burns Iris hemorrhage from site of laser peripheral iridotomy (with Nd:YAG) – applying pressure on the globe with the laser lens is usually sufficient to stop the hemorrhage	
	IOP spike	
	Anterior chamber inflammation with closure of iridotomy, formation of posterior synechiae or raised IOP	
	Cataract formation	
	Corneal endothelial decompensation, malignant glaucoma, retinal damage, cystoid macular edema (all rare)

**Box 3:** Classification Based on Anatomic Levels of Obstruction to Aqueous Flow (Pathophysiology)

**Figure 1:**
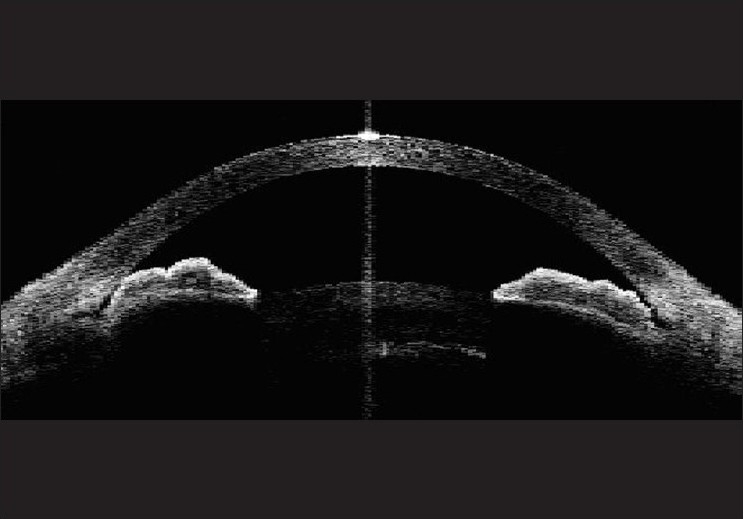
Anterior segment optical coherence tomography image demonstrating pupillary block mechanism

Apposition of the iris to the trabecular meshwork in ACG may be due to forces acting at four anatomic levels as follows.
Iris
Pupillary block [Fig. 1]
*Non-pupil block/angle crowding mechanisms, e.g., thick peripheral iris roll [Figs. 2a, b]
Contraction of fibrvascular membrane in neovascular glaucoma
Contraction of fibrin in angle secondary to anterior uveitis or hyphema
Endothelial proliferation (iridocorneoendothelial syndromes)
*Epithelial downgrowth*
Ciliary body
Plateau iris configuration (forward rotation of the ciliary body (CB) or anterior position of ciliary processes) [Fig. 3]
*Ciliary body cysts (pseudoplateau iris)*
Lens
Phacomorphic glaucoma (thick lens)
Phakotopic glaucoma (anteriorly positioned lens)
*Subluxed lens (e.g., pseudoexfoliation syndrome, traumatic)*
Vectors posterior to lens
Aqueous misdirection (malignant glaucoma)
Serous or hemorrhagic choroidal detachment or effusion
Space-occupying lesion (gas bubble, vitreous substitutes, tumor)
Retrolenticular tissue contracture (retinopathy of prematurity, persistent hyperplastic primary vitreous)
(Secondary causes of angle closure are shown in italics.)
Each level of block may have a component of each of the levels preceding it, and some patients may have a combination of multiple mechanisms.

* Non-pupil block/angle crowding mechanisms have been included here, as an addition to Ritch’s classification.

**Figure 2a:**
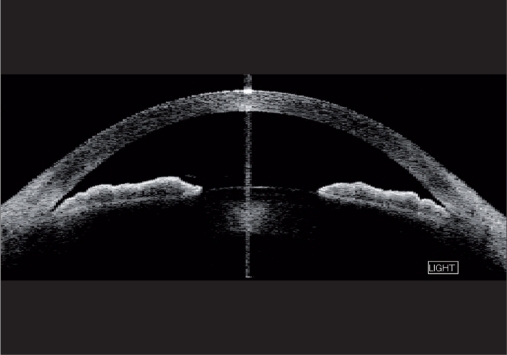
Anterior segment optical coherence tomography images of the same eye taken in light

**Figure 2b:**
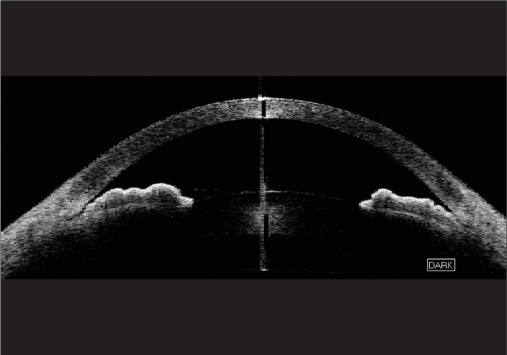
Anterior segment optical coherence tomography images of the same eye taken in dark conditions demonstrating peripheral iris roll

**Figure 3:**
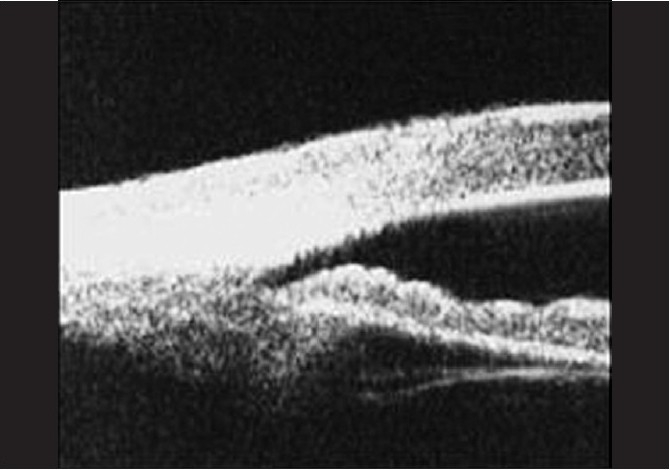
Ultrasound biomicroscopy image demonstrating plateau iris configuration

**Figure 4:**
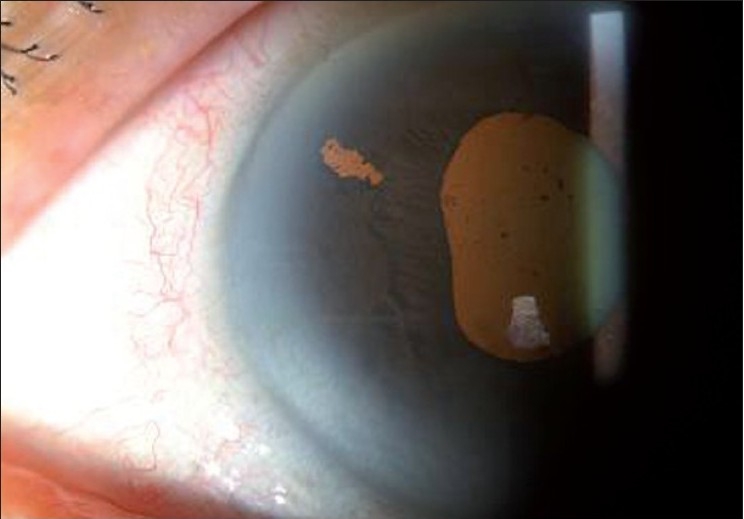
Anterior segment photograph showing laser peripheral iridotomy in acute angle closure eye

### Laser iridoplasty

**Figure 5:**
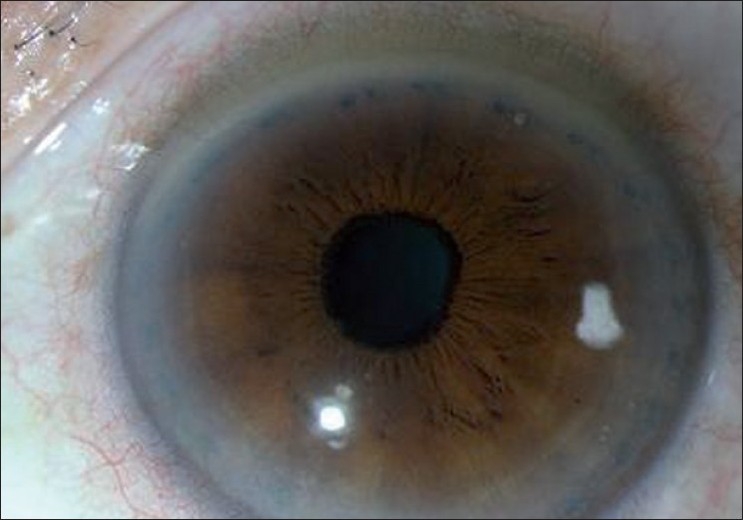
Anterior segment photograph showing laser iridoplasty marks

Where mechanisms other than pupillary block exist, laser peripheral iridotomy may be insufficient in opening the anterior chamber angle. The Liwan Eye Study found residual iridotrabecular contact in 59% of Chinese eyes after successful laser peripheral iridotomy and attributed this to smaller anterior chamber angle dimensions and thicker irides.[16] In such cases, argon laser peripheral iridoplasty [Fig. 5] has been found to be effective. By applying surface photocoagulation burns in the iris, tissue contraction results in pulling of the peripheral iris away from the trabecular meshwork, thereby opening the anterior chamber angle.

**Table:** 

**Indications:**	Appositional angle closure with or without peripheral anterior synechiae or elevated IOP
	Plateau iris configuration
	Where angle remains appositionally closed or occludable following laser peripheral iridotomy
	Thick peripheral iris roll
	In acute AC, to help break the attack where medical therapy has failed or is contraindicated
	To facilitate access to trabecular meshwork for laser trabeculoplasty
**Procedure:**	Abraham/Wise/Goldmann 3-mirror lens
	Aim at iris as peripheral as possible, outside of any corneal arcus senilis
	Argon green or blue-green, or diode 200–500 mW, 100–200 µm spot size, 0.2–0.5 seconds, single row of about 25 burns over 360°
	Endpoint: Iris stromal contraction accompanied by progressive peripheral anterior chamber deepening with increasing number of burns
**Complications:**	Corneal endothelial burns
	Iritis
	IOP spike
	Peripheral anterior and/or posterior synechiae

Studies are ongoing to find the long-term efficacy of laser iridoplasty.

## Surgical Management

Surgical management is indicated where there is inadequate control of the IOP with progression of optic nerve or visual field damage despite medical and laser treatment. Early lens extraction has been advocated, especially where there is a significant cataract that is impairing vision. If performed prior to the formation of peripheral anterior synechiae, it may prevent progressive AC. Other indications for early surgical management include poor compliance or intolerance to medical treatment and inability to cooperate with laser treatment.

## Iridectomy

Since the advent of laser iridotomy, surgical peripheral iridectomy is now seldom performed. However, it may still be useful occasionally where the cornea fails to clear sufficiently for laser iridotomy to be performed, or in the case of a patient who is unable to cooperate with the laser procedure. A 2–3 mm partial-thickness incision (to about two-thirds of the corneal thickness) is made, usually in the superotemporal peripheral cornea. The incision may also be made at the limbus after a limited conjunctival peritomy. A nylon suture is placed and looped out of the incision groove. An assistant may use this suture to open or close the incision later so as to control the rate of aqueous egress. The anterior chamber is then entered with the blade held vertically. A knuckle of iris should prolapse into the wound, if necessary, with some counter-pressure on the posterior lip of the incision. A toothed forceps is used to hold the prolapsed iris, and Vannas scissors are used to excise it. Neither forceps nor scissors enter the anterior chamber, thus avoiding any risk of damage to the lens or other structures. The edges of the incision are then stroked to encourage the iris to retract into the anterior chamber, and the corneal incision is closed with one or two 10-0 nylon sutures.

### Lens extraction

Removal of the lens, especially if there is any evidence of cataract, is helpful particularly where either the lens thickness or its anterior position is thought to be the main mechanism underlying angle closure. However, if done in the acute scenario, care must be taken during surgery as these eyes are usually associated with high IOPs, shallow anterior chambers, cloudy cornea, decreased corneal endothelial cell counts, floppy iris due to previous ischemia, posterior synechiae, bulky lens, lax lens zonules, and a high risk of malignant glaucoma. Reports of phacoemulsification combined with goniosynechialysis, in the presence of peripheral anterior synechial closure, have been encouraging.[17–19] However, there is as yet no evidence from good-quality randomized trials or nonrandomized studies of the effectiveness of lens extraction for chronic PACG.[20]

### Goniosynechialysis

This is usually performed in combination with lens extraction and involves mechanical stripping of peripheral anterior synechiae away from the trabecular meshwork, using viscoelastics or an irrigation cyclodialysis spatula.[17,18]

### Trabeculectomy

Trabeculectomy in ACG is performed similarly as for open angle glaucoma, with the exception that a surgical peripheral iridectomy should always be performed at the time of trabeculectomy in the former. In addition, the use of antimetabolites should be considered. Trabeculectomy, either alone or in combination with lens extraction, should be considered after the acute attack of AC, if the IOP control remains suboptimal despite laser and medical treatment, especially in more advanced cases of ACG which are already associated with peripheral anterior synechiae, optic nerve or visual field damage. In acutely inflamed eyes, trabeculectomy has been reported to have low success rates.[21]

### Glaucoma drainage implant

This may be considered in chronic ACG where trabeculectomy has failed to control the IOP, or in eyes that are deemed to be at high risk of failure with trabeculectomy.

## Conclusion

Angle closure can be associated with good visual prognosis, provided it is detected early and the appropriate treatment given. Also, 42–72% of cases presenting with acute PAC can be satisfactorily treated with laser iridotomy alone,[22,23] and 60–75% of such patients recover without optic disc or visual field damage, if the IOP is promptly and adequately controlled.[24] However, a longer duration of the angle closure attack or a history of intermittent angle closure episodes is often associated with the need for additional medical or even surgical therapy.[25–27] The presence of a significant amount of peripheral anterior synechiae, a higher presenting IOP and a larger cup:disc ratio on presentation are other predictors of inadequate pressure control despite a patent laser peripheral iridotomy.[28–30] Most patients who develop a rise in IOP after laser peripheral iridotomy do so within the first 6 months.[27] Once glaucomatous optic neuropathy and visual field damage have developed, 94–100% may require further surgical treatment to control IOP.[31]

Perhaps one of the most important and cost-effective methods of managing PACG in the population currently is by increasing public awareness of the disease so that patients at risk, such as those with a positive family history or with ocular risk factors, can undergo risk assessment and prophylactic treatment where necessary. Along with this, better imaging devices and sound population screening strategies will go a long way to help identify others at risk and thus help to reduce visual morbidity due to this disease.

## References

[1] Resnikoff S, Pascolini D, Etya’ale D, Kocur I, Pararajasegaram R, Pokharel GP (2004). Global data on visual impairment in the year 2002. Bull World Health Organ.

[2] Quigley HA, Broman T (2006). The number of people with glaucoma worldwide in 2010 and 2020. Br J Ophthalmol.

[3] Foster PJ, Buhrmann RR, Quigley HA, Johnson GJ (2002). The definition and classification of glaucoma in prevalence surveys. Br J Ophthalmol.

[4] Ritch R, Lowe RF (1996). Angle closure glaucoma. The Glaucomas.

[5] Chew PT, Aung T, EXACT Study Group (2004). Intraocular pressure-reducing effects and safety of latanoprost versus timolol in patients with chronic angle closure glaucoma. Ophthalmology.

[6] Sihota R, Saxena R, Agarwal HC, Gulati V (2004). Crossover comparison of timolol and latanoprost in chronic primary angle closure glaucoma. Arch Ophthalmol.

[7] Agarwal HC, Gupta V, Sihota R (2003). Effect of changing from concomitant timolol pilocarpine to bimatoprost monotherapy on ocular blood flow and IOP in primary chronic angle closure glaucoma. J Ocul Pharmacol Ther.

[8] Aung T, Chan YH, Chew PT, EXACT Study Group (2005). Degree of angle closure and the intraocular pressure-lowering effect of latanoprost in subjects with chronic angle closure glaucoma. Ophthalmology.

[9] Kook MS, Cho HS, Yang SJ, Kim S, Chung J (2005). Efficacy of latanoprost in patients with chronic angle closure glaucoma and no visible ciliary body face: A preliminary study. J Ocul Pharmacol Ther.

[10] Chew PT, RojanaPongpun P, Travatan CACG Study Group (2006). Travatan CACG Study Group. Intraocular pressure-lowering effect and safety of Travoprost 0.004% and Latanoprost 0.005% for the treatment of chronic angle closure glaucoma. Asian J Ophthalmol.

[11] Aung T, Chew PT (2002). Review of recent advancements in the understanding of primary angle-closure glaucoma. Curr Opin Ophthalmol.

[12] Bessette FM, Nguyen LC (1989). Laser light: Its nature and its action on the eye. CMAJ.

[13] Liebmann JM, Ritch R (2002). Laser surgery for angle closure glaucoma. Semin Ophthalmol.

[14] Ho T, Fan R (1992). Sequential argon-YAG laser iridotomies in dark irides. Br J Ophthalmol.

[15] See J, Chew PT (2006). Angle Closure Glaucoma. Yanoff and Duker Ophthalmology. Chapter 10.

[16] He M, Friedman DS, Ge J, Huang W, Jin C, Cai X (2007). Laser peripheral iridotomy in eyes with narrow drainage angles: Ultrasound biomicroscopy outcomes. The Liwan Eye Study. Ophthalmology.

[17] Harasymowycz PJ, Papamatheakis DG, Ahmed I, Assalian A, Lesk M, Al-Zafiri Y (2005). Phacoemulsification and goniosynechialysis in the management of unresponsive primary angle closure. J Glaucoma.

[18] Teekhasaenee C, Ritch R (1999). Combined phacoemulsification and goniosynechialysis for uncontrolled chronic angle closure glaucoma after acute angle closure glaucoma. Ophthalmology.

[19] Tham CC, Leung DY, Kwong YY, Li FC, Lai JS, Lam DS (2010). Effects of Phacoemulsification Versus Combined Phaco-trabeculectomy on Drainage Angle Status in Primary Angle Closure Glaucoma (PACG). J Glaucoma.

[20] Friedman DS, Vedula SS (2006). Lens extraction for chronic angle-closure glaucoma. Cochrane Database Syst Rev.

[21] Aung T, Tow SL, Yap EY, Chan SP, Seah SK (2000). Trabeculectomy for acute primary angle closure. Ophthalmology.

[22] Aung T, Ang LP, Chan SP, Chew PT (2001). Acute primary angle closure: Long-term intraocular pressure outcome in Asian eyes. Am J Ophthalmol.

[23] Playfair TJ, Watson PG (1979). management of acute primary angle closure glaucoma: A long-term follow-up of the results of peripheral iridectomy used as an initial procedure. Br J Ophthalmol.

[24] Dhillon B, Chew PT, Lim AS (1990). Field loss in primary angle closure glaucoma. Asia-Pac J Ophthalmol.

[25] Buckley SA, Reeves B, Burdon M, Moorman C, Wheatcroft S, Edelsten C (1994). Acute angle closure glaucoma: Relative failure of YAG iridotomy in affected eyes and factors influencing outcome. Br J Ophthalmol.

[26] Saunders DC (1990). Acute closed angle glaucoma and Nd: YAG laser iridotomy. Br J Ophthalmol.

[27] Alsagoff Z, Aung T, Ang LP, Chew PT (2000). Long-term clinical course of primary angle closure glaucoma in an Asian population. Ophthalmology.

[28] Aung T, Lim MC, EXACT Study Group (2005). Configuration of the drainage angle, intraocular pressure and optic disc cupping in subjects with chronic angle closure glaucoma. Ophthalmology.

[29] Salmon JF (1993). Long-term intraocular pressure control after Nd: YAG laser iridotomy in chronic angle closure glaucoma. J Glaucoma.

[30] Nolan WP, Foster PJ, Devereux JG, Uranchimeg D, Johnson GJ, Baasanhu J (2000). YAG laser iridotomy treatment for primary angle closure in east Asian eyes. Br J Ophthalmol.

[31] Rosman M, Aung T, Ang LP, Chew PT, Liebmann JM, Ritch R (2002). Chronic angle closure with glaucomatous damage: Long-term clinical course in a North American population and comparison with an Asian population. Ophthalmology.

